# Gamification of Behavior Change: Mathematical Principle and Proof-of-Concept Study

**DOI:** 10.2196/43078

**Published:** 2024-03-22

**Authors:** Falk Lieder, Pin-Zhen Chen, Mike Prentice, Victoria Amo, Mateo Tošić

**Affiliations:** 1 Department of Psychology University of California, Los Angeles Los Angeles, CA United States; 2 Max Planck Institute for Intelligent Systems Tübingen Germany

**Keywords:** gamification, points, feedback, behavior change, habit formation, chatbot, digital interventions, mobile phone, artificial intelligence

## Abstract

**Background:**

Many people want to build good habits to become healthier, live longer, or become happier but struggle to change their behavior. Gamification can make behavior change easier by awarding points for the desired behavior and deducting points for its omission.

**Objective:**

In this study, we introduced a principled mathematical method for determining how many points should be awarded or deducted for the enactment or omission of the desired behavior, depending on when and how often the person has succeeded versus failed to enact it in the past. We called this approach *optimized gamification of behavior change*.

**Methods:**

As a proof of concept, we designed a chatbot that applies our optimized gamification method to help people build healthy water-drinking habits. We evaluated the effectiveness of this gamified intervention in a 40-day field experiment with 1 experimental group (n=43) that used the chatbot with optimized gamification and 2 active control groups for which the chatbot’s optimized gamification feature was disabled. For the first control group (n=48), all other features were available, including verbal feedback. The second control group (n=51) received no feedback or reminders. We measured the strength of all participants’ water-drinking habits before, during, and after the intervention using the Self-Report Habit Index and by asking participants on how many days of the previous week they enacted the desired habit. In addition, all participants provided daily reports on whether they enacted their water-drinking intention that day.

**Results:**

A Poisson regression analysis revealed that, during the intervention, users who received feedback based on optimized gamification enacted the desired behavior more often (mean 14.71, SD 6.57 times) than the active (mean 11.64, SD 6.38 times; *P*<.001; incidence rate ratio=0.80, 95% CI 0.71-0.91) or passive (mean 11.64, SD 5.43 times; *P*=.001; incidence rate ratio=0.78, 95% CI 0.69-0.89) control groups. The Self-Report Habit Index score significantly increased in all conditions (*P*<.001 in all cases) but did not differ between the experimental and control conditions (*P*>.11 in all cases). After the intervention, the experimental group performed the desired behavior as often as the 2 control groups (*P*≥.17 in all cases).

**Conclusions:**

Our findings suggest that optimized gamification can be used to make digital behavior change interventions more effective.

**Trial Registration:**

Open Science Framework (OSF) H7JN8; https://osf.io/h7jn8

## Introduction

### Background

#### Overview

People often struggle to change their behavior in ways that would benefit them in the long run. For instance, many people could improve their health and life expectancy by building healthy habits such as exercising [1], healthy eating [2], or drinking a glass of water before every meal [3,4]. People who want to adopt healthy habits because they know about their benefits nevertheless struggle to change their behavior accordingly.

Building a good habit is especially difficult when the benefits of the desired behavior cannot be felt immediately. One approach to alleviate this challenge is to create apps that encourage the desired behavior using incentives or immediate positive feedback [5-7] or discourage unwanted behavior using immediate negative feedback [8]. Doing so in a gameful way can be a promising approach to improving people’s health behaviors [5-7,9-12]. Using game elements to improve people’s behavior in the real world is known as gamification [13]. One of the most commonly used gamification methods is awarding people points for specific behaviors [14,15]. The awarded points are often used to provide feedback to the user, define levels, award badges, or create leaderboards.

Despite the widespread use of points, levels, badges, and leaderboards, there is currently no principled way to choose precisely how many points a person should be awarded and when. This is a problem as making those decisions based on intuition can lead to point systems that inadvertently incentivize counterproductive behaviors or undermine the users’ motivation [16-18]. For instance, a recent study found that the point system of the popular gamified habit formation app Habitica is actively harmful [17].

To help practitioners master the challenge of designing effective point systems that reliably foster positive behavior change, we introduced a mathematical principle for computing the number of points a person should receive for engaging in the desired behavior depending on their history of either engaging or not engaging in this behavior and how many points they should lose when they fail to do so. We called this principle *optimized gamification of behavior change*. As a proof of concept, we applied optimized gamification to design a chatbot that helps people develop the healthy habit of drinking water before every meal [3,4]. Our chatbot awards points for the desired behavior and deducts points for its omission. Critically, the number of points that the user gains or loses is computed using optimized gamification. Our chatbot combines optimized gamification with three established principles of behavior change: it (1) guides the user to set an implementation intention, (2) reminds them of their good intentions, and (3) supports self-monitoring.

We evaluated our chatbot in a longitudinal field experiment and found that optimized gamification can make digital behavior change interventions more effective. Our findings provide a proof of concept for a very general and principled approach to improving human behavior in the real world. In addition to this theoretical contribution, we introduced a chatbot for helping people develop a specific healthy habit (water drinking): the Good Habit Bot. This chatbot can easily be adapted to other health behaviors, including more critical health behaviors such as exercising, healthy eating, and other good habits that people want to establish.

The plan for this paper is as follows. We first introduce relevant background information about behavior change and gamification. We then present our theory of optimized gamification for behavior change. Next, we present the chatbot we designed as a proof of principle. After that, we present the methods and results of our field experiment and discuss its findings and implications.

#### Behavior Change Goals Versus Automatic Behavior

Human behavior is controlled by a combination of goal-directed decision-making (eg, *I will buy a gym membership because I want to lose weight*) and more automatic reactions to certain stimuli (eg, always stopping by the gym on the way home from work) [19]. Goal-directed decision-making derives choices from the outcomes that people value (eg, health or money) or want to avoid (eg, pain) according to their mental models of how those outcomes can be obtained. In contrast, automatic reactions do not consider the behavior’s likely consequences in the current situation.

#### Obstacles to Behavior Change: Automaticity and Temporal Discounting

Most of our behavior is not primarily controlled by goal-directed decision-making but determined by people’s automatic reactions. Therefore, automatic behavioral responses can interfere with people’s ability to act in accordance with their behavior change goals. This is the proverbial conflict between bad habits and good intentions. People can inhibit and override their automatic behavioral responses (*bad habits*)*,* but their capacity to do so is limited [20]. Therefore, the automaticity of human behavior is a crucial obstacle to intentional behavior change.

A second obstacle to successful behavior change is that the mechanisms of goal-directed decision-making are demonstrably biased in favor of immediate outcomes [21]. People give too much weight to their decision’s immediate consequences and too little weight to its long-term consequences. This phenomenon is known as present bias [22]. It has been proposed that present bias occurs because the brain discounts delayed benefits as if they become less valuable the later they occur [23]. This phenomenon, which is known as temporal discounting, is well established in research on economic decisions and animal behavior.

Moreover, according to temporal motivation theory [24], temporal discounting is one of the main reasons why people fail to enact good intentions. Such failures of self-regulation are a critical obstacle to health behavior change [25,26]. Consistent with this explanation, people who discount delayed outcomes more strongly are likelier to engage in unhealthy behaviors and experience poor health [26].

#### Reinforcement Learning as a Mechanism of Behavior Change

As automatic responses are powerful drivers of human behavior, successful behavior change typically involves translating behavior change goals into automatic behavioral responses [27].

Automatic behavioral responses, including exercise habits, can be acquired through learning from experience. Model-free reinforcement learning is a well-established mechanism of learning automatic behavioral responses from experience [19]. This mechanism increases or decreases a person’s propensity to engage in a specific behavior in a particular situation (eg, going to the gym after work) according to whether they experience the behavior’s overall consequences as positive or negative. The vast literature on operant conditioning in animals and humans underscores that learning from reward and punishment is a powerful mechanism of behavior change [28,29]. Another complementary learning mechanism involves strengthening habits through mere repetition [30].

#### Supporting Behavior Change Through Incentives and Reinforcement

The literature surveyed previously demonstrates that goal-directed decision-making and automatic behavioral responses are responsive to rewards and punishments. Goal-directed decision-making is sensitive to anticipated future rewards, and automatic behavioral responses are shaped by the rewards or punishments that those behaviors have generated in the past. These effects can be leveraged to support behavior change. To foster behavior change via goal-directed decision-making, behavior change interventions can create and announce incentives for engaging in the desired behavior. To foster behavior change via reinforcement learning, behavior change interventions can reinforce the desired behavior with rewards or positive feedback.

A highly effective behavior change intervention that leverages both effects is contingency management [31]. Contingency management incentivizes a desired behavior change and rewards people when they enact it. Voucher-based reinforcement therapy for treating addiction is a highly successful example of contingency management [32]. This behavior change intervention awards the patient a voucher every time they submit a negative drug test. More recently, it has also been applied to foster other types of behavior change, including physical exercise [7,33] and treatment attendance [34]. Contingency management appears to be more effective when the desired behavior is reinforced more promptly, more frequently, and with rewards that are larger or increase throughout the intervention [35-37].

#### Digital Behavior Change Interventions

Developing digital behavior change interventions is a young and booming field [38]. Mobile apps have shown potential for fostering positive behavior change in domains such as physical exercise and healthy eating [38,39]. However, the average effect size of such interventions is still relatively small [40,41]. Most behavior change interventions are not derived from any theory, model, or framework [38]. Therefore, we suspect that there is still room for improvement and that at least some of this potential can be realized by adopting a more theory-driven approach.

Goal setting and self-monitoring are the most commonly used behavior change techniques [38]. A meta-analysis of studies on digital interventions for promoting physical exercise found that these 2 techniques are also the most effective ingredients of current digital behavior change interventions [41]. Goal setting entails guiding people to articulate their intent to perform certain behaviors in certain situations (eg, drinking a glass of water before every meal). Supporting self-monitoring often takes the form of helping people check or record whether they have enacted those intentions or track related outcomes (eg, their weight). Goal setting is especially effective when people formulate simple plans that specify the intended behavior and the situation in which they want to perform it as concretely as possible [42,43]. This approach is known as *implementation intentions*. Moreover, reminding people of their intentions via SMS text messages [44] and presenting them with positive reinforcement when they enact their intentions [5,7,33] have been found to be highly effective in promoting physical exercise.

#### Gamification

A recent meta-analysis found that approximately 1 in 5 digital behavior change interventions are designed within the gamification framework [38]. Gamification entails applying principles from game design and game elements, such as storytelling and rules for earning points and winning the game, to address real-world problems [13]. The basic idea is to motivate people to do things that benefit them or others, such as exercising and studying, in a gameful way. Previous research has found that gamification can improve people’s behavior, achieve desired outcomes, and improve people’s subjective experiences [45].

Gamification is already widely used in designing digital behavior change interventions [5], and previous studies have suggested that it can improve people’s health behaviors [7,9-12,33]. One gamification strategy that is effective in digital behavior change interventions is awarding the user points as positive feedback for the desired behavior [5,7,33]. Such extrinsic incentives can increase the frequency of the targeted behaviors without affecting people’s intrinsic motivation [46,47].

However, when gamification is not correctly designed, it can backfire and have adverse effects [16,17,48]. This has also been observed in the behavior change literature [12,49] and in gamified habit formation apps [17]. Getting the incentives exactly right can be crucial as points, levels, badges, and leaderboards do not foster the user’s intrinsic motivation [46,47] and might sometimes even undermine it when they are not embedded in a compelling narrative [18]. Motivated by these problems, many authors have called for a more theory-driven approach to gamification in general [50] and gamifying digital behavior change interventions in particular [12].

#### Optimized Gamification

Previous work has investigated how many points should be awarded for which behavior to maximally benefit the user in the context of to-do list apps that help the user achieve their own goals [51,52]. Building on temporal motivation theory [24], this work assumed that people’s motivation is insufficiently sensitive to long-term benefits such as good health in old age and overly sensitive to immediate costs (eg, the effort of exercising) and short-term pleasure (eg, from receiving immediate positive feedback). To help people overcome the resulting motivational problems (eg, procrastination) [53], Lieder et al [51] developed a mathematical theory for designing point systems that provide immediate positive feedback for activities that are beneficial in the long run and immediate negative feedback for activities that are not. The basic idea is to align each action’s immediate and long-term consequences. The action that is best in the long run should be made most appealing in the short run, and actions with undesirable long-term consequences should be made unappealing in the short run.

Therefore, optimized gamification strives to incentivize each of the available actions through a number of points proportional to how much that action increases or decreases the sum total of future happiness. This idea is implemented by modeling the activities to be incentivized as steps that lead toward a valuable goal or away from it. Actions that lead toward the goal increase the time the person will spend in the more valuable state in which the goal has been achieved. In contrast, actions that lead away from the goal decrease the time the person will spend in the more valuable state in which the goal has been achieved and increase the effort required to achieve it afterward. On the basis of this mathematical model, dynamic programming and reinforcement learning methods can estimate how much a given action improves or worsens the person’s situation. These estimates are then translated into incentives that encourage good choices and discourage bad ones. The resulting point values are optimal in that they would enable even a purely myopic decision maker who only cares about immediate outcomes to choose the actions that are best for them in the long run [51].

Although optimized gamification construes points as incentives and uses mathematical and computational methods from the field of reinforcement learning, using it does not constitute a commitment to behaviorism and is fully compatible with cognitive theories of motivation [54].

Optimized gamification has been used to encourage users to tackle the tasks on their to-do lists [32] and encourage students to select the most valuable learning activities [55,56]. Optimized gamification has also been applied to give people feedback on how they think about what to do [57] and on whether they succeeded in staying focused on a chosen task or got distracted [58]. However, to date, this approach has never been applied to support habit formation.

### Objectives

The first goal of this study was to introduce a principled method for computing feedback on the enactment or omission of the desired behavior and experimentally test whether it can be used to enhance digital behavior change interventions. The second goal of this study was to introduce a chatbot that uses this method to help people develop healthy water-drinking habits and evaluate it in a longitudinal field experiment.

## Methods

### Optimized Gamification of Behavior Change

#### Overview

We conceptualized behavior change as a special case of repeatedly choosing and learning when to do what. As reviewed in the *Background* section, optimized gamification can encourage desired behaviors and accelerate learning [57]. To apply this method to promote the desired behavior and accelerate the formation of healthy habits, we first have to model habit formation as a Markov decision process (MDP) [59].

#### Modeling Habit Formation

An MDP is a scenario in which an agent faces a series of choices. Each choice (*a*) has 2 effects. First, it yields an immediate reward (*r*) that may be positive, negative, or zero. Second, it may change the state (*s*) the agent finds itself in. In an MDP, the agent’s goal is to maximize the sum of the rewards it accumulates from its first decision to its last one.

We model behavior change problems as a straightforward MDP, in which a person repeatedly chooses between 2 possible actions when they find themselves in a particular situation: enacting the desired behavior (*a*=1) or not enacting it (*a*=0). Our model assumes that a given behavior change intervention aims to turn the desired behavior into a habit. Therefore, we define the state as the strength of the person’s healthy habit, measured using a single number, *s*_habit_, which can range from 0 to 1. Following standard habit formation models [30], we assume that enacting the habit increases its strength from

*s*_habit_ to *s*_habit_ + α × (1 – *s*_habit_),

where α is a free parameter that describes how quickly habits form. Conversely, our model assumes that failure to enact the desired behavior decreases the strength of the habit to *s*_habit_ × (1 – α). We assume that the habit has been cultivated when its strength exceeds some threshold θ (eg, θ=0.9) and model the health benefits conferred by achieving this goal as a reward (*r*_goal_) that is attained when the habit strength crosses this threshold. Enacting the desired behavior is assumed to incur a cost that decreases with the strength of the habit (*r*[*s*_habit_, 1]=–[1 – *s*_habit_]), whereas not performing the behavior is assumed to be effortless (*r*[*s*_habit_, 0]=0).

#### Computing Optimal Feedback

The basic idea of optimized gamification is to reward each action using a number of points that are proportional to its long-term benefits. These long-term benefits are measured via the decrease in future costs and the increase in future rewards brought about by transitioning to a state in which the habit is stronger. In situations in which the benefits of developing the good habit outweigh its costs, the value of having a habit of strength *s*_habit_ and then following through with the process of building the habit is as follows:




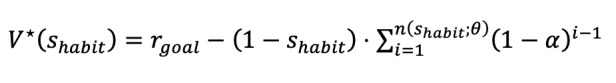




where the number *n*(*s*_habit_; θ) specifies how often the behavior must be enacted until the habit strength reaches its target value θ. Therefore, for someone who will follow through with building the habit, the long-term benefits of enacting the habit one more time when its current strength is *s*_habit_ are

*f*(*s*_habit_, 1) = V*(*s*_habit_ + α × [1 – *s*_habit_]) – V*(*s*_habit_) = 1 – *s*_habit_.

Conversely, the long-term costs of failing to enact the desired behavior in the situation in which it is supposed to become a habit are

*f*(*s*_habit_, 0) = V*(*s*_habit_ × [1 – α]) – V*(*s*_habit_).

Please note that, even though we are talking about a situation in which it is rational for people to build the habit, this does not mean that we assume people to be rational. For our method, it does not matter why people follow through with building the habit. In fact, we assume that some people will follow through with building the habit only because they are (irrationally strongly) motivated by the immediate rewards conferred by feedback.

The lowest possible negative value of *f* is *f*(θ, 0), and the largest possible positive value is *f*(0, 1). Although the exact values depend on the model parameters, they are typically approximately –1 and 1, respectively. Therefore, to transform those values into points, it is desirable to scale them by the desired maximum point value (*M*) that the application should award to the user and then round the scaled values to the nearest integer. This yields the following equation for the number of points that the application should award when a user reports that they have enacted their intention (*a*=1) or have not enacted their intention (*a*=0):

points(*s*_habit_, *a*) = round(*M* × *f*(*s*_habit_, *a*)) (Equation 1).

**Figure 1:**
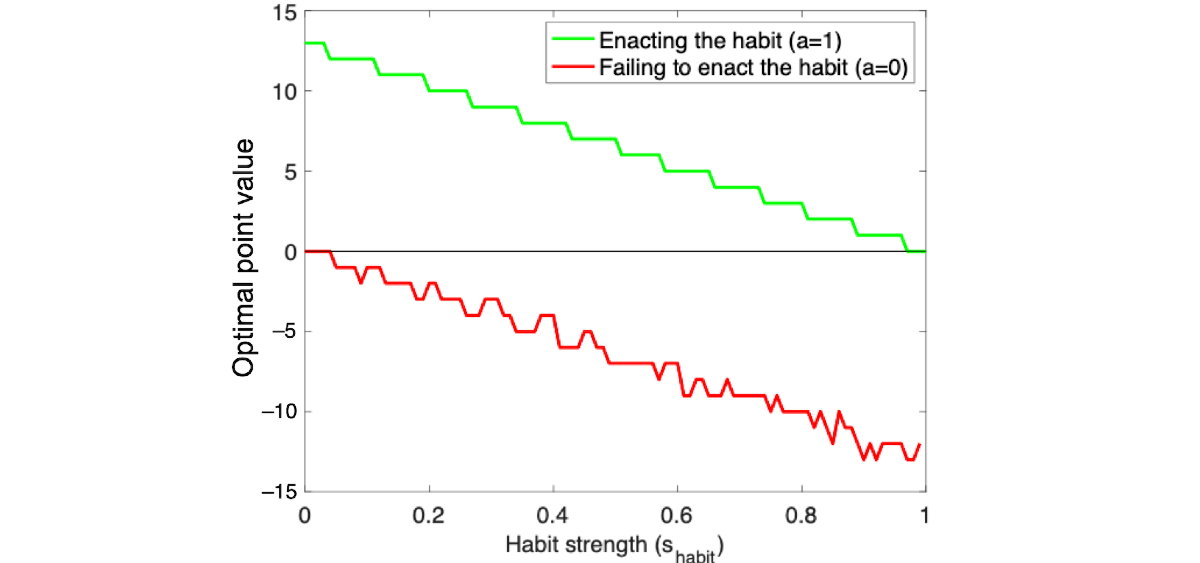
Point values for enacting versus failing to enact the habit at different habit strengths for a learning rate of α=0.1 and a threshold of θ=0.9.

Figure 1 illustrates the point values as a function of the user’s habit strength in an example application with a learning rate of α=0.1, a target habit strength of θ=0.9, and a maximal point value of *M*=13. Multimedia Appendix 1 provides more details on our mathematical model and the resulting optimal point system.

As illustrated in Figure 1, the number of points for enacting the desired behavior is largest when the habit is weakest and gradually decreases toward 0 as the habit strengthens. This is intuitive as performing the desired behavior becomes easier the more often one has already performed it. Conversely, the number of points that should be deducted when the user fails to enact the habit is smallest when the strength of the habit is 0 and then increases as the habit becomes stronger. This is an intuitive consequence of our model’s assumption that failing to enact the desired behavior in the specified situation weakens the habit strength by approximately 10%. The stronger the habit, the more is lost when its strength is reduced by 10%. The number of points awarded for enacting the desired behavior is a monotonically decreasing function of the habit strength. In contrast, the point value for failing to enact the desired behavior changes more erratically. This is because the number of steps required to reach the desired habit strength changes abruptly with the current habit strength. For instance, failing to enact the behavior at a habit strength of 0.09 increases the number of times the behavior needs to be enacted to achieve the desired habit strength from 21 to 22 times. However, if the habit strength is 0.08 or 0.10, the number of times the desired behavior has to be enacted remains 22 and 21 times, respectively. Individual users rarely experience such irregular changes as the change in their habit strength typically skips across those small areas in which the point value changes nonmonotonically. Moreover, our simulations suggest that the penalty for failing to enact the desired behavior can be approximated using a linear function with the same slope as the number of points for performing the desired behavior.

#### Application to Supporting Positive Behavior Change

The optimized gamification method described previously can be applied to help people form good habits. The equations for computing the number of points are easy to implement within digital behavior change interventions such as chatbots and habit trackers. All that is needed is to ask the user which habit they want to develop and estimate its initial strength, set the learning rate parameter and the habit’s target strength to reasonable values (eg, α=0.1 and θ=0.9), and record when the user did versus did not act in accordance with the desired habit. To define the desired habit, the user has to specify the desired behavior and the situation in which they want to perform it. The user’s initial habit strength can be estimated through the desired behavior’s relative frequency in that situation in the previous weeks (eg, at 2 of the previous 7 lunches). The habit strength can then be initialized using that proportion. Alternatively, when it makes sense to assume that the user wants to build an entirely new habit, the strength can be initialized as 0. Then, whenever the user reports having or not having enacted their intention, the optimized gamification equation can be applied to compute how many points the user should gain or lose. Whenever the user reports having performed the desired behavior, the estimate of the habit strength should be increased to

*s*_habit_ + α × (1 – *s*_habit_),

and whenever the user reports having missed an opportunity to enact their intention, the estimate of their habit strength decreases to *s*_habit_ × (1 – α). Then, the same procedure repeats when the user reports on their next opportunity to enact the behavior.

This approach can be used to support many different types of positive behavior change. It can help people build good habits in areas such as exercise, sleep, taking medication, nutrition, work, chores, and leisure activities. It can be used in interventions focusing on specific habits and, in general, in habit formation tools that let users choose any habit they want to develop. Another possible application is helping people overcome bad habits (eg, smoking cessation) [60]. It can support applications that run on practically any device, from smartwatches and wristbands to mobile phones, desktop applications, web applications, and smart glasses.

### Proof of Concept: A Chatbot for Building a Healthy Water-Drinking Habit

As a proof of concept for the application of optimized gamification to support behavior change, we implemented this idea as a Telegram (Telegram FZ LLC) chatbot called the *Good Habit Bot*. This chatbot helps the user develop a healthy water-drinking habit by combining 4 behavior change techniques: goal setting, reminders, support for self-monitoring, and feedback. Concretely, the Good Habit Bot guides the user to formulate an implementation intention that links a specific desired behavior to a concrete daily event, reminds the user of their intention on a daily basis, checks in with them on whether they followed through on their intention every day, and then gives them positive or negative feedback depending on whether they did or did not follow through on their intention.

**Figure 2:**
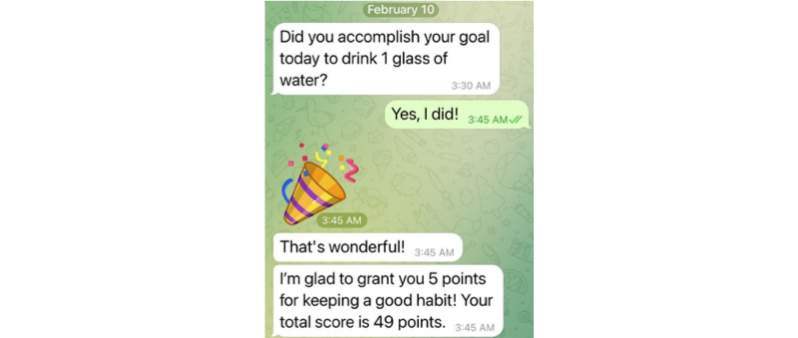
Screenshot of the feedback method with point values computed by our optimized gamification method.

When the user starts their first conversation with the Good Habit Bot, the chatbot introduces itself and says that its purpose is to help the user form a healthy water-drinking habit. The Good Habit Bot then asks the user to choose which of 8 concrete, recurring moments in their day they want to use as the trigger for their water-drinking habit (eg, *when my wake-up alarm rings* or *when I have the first bite of my lunch*; for the complete list, see Multimedia Appendix 2). Next, the Good Habit Bot asks the user how much water they want to drink in that situation (eg, 1 glass or 0.5 glasses) and how often they did so in the previous 7 days. The chatbot then uses the number of days *n* of the previous week in which the user acted in accordance with the habit (eg, n=2) to initialize their habit strength by n/7 (eg, *s*_habit_ = 2/7). Then, in the evening of the first day (ie, at 9 PM), the chatbot reminds the user of their intention to drink a specific amount of water at a particular moment of the following day (eg, *Remember your intention*: *When I have the first bite of my lunch, I will drink 1.5 glasses of water*). Then, sometime after the moment in which the user wanted to enact their intention, the chatbot asks them whether they did so (ie, *Did you accomplish your goal today to drink 1.5 glasses of water*). If the user affirms that they followed through on their intention, the chatbot gives them positive feedback (Figure 2). This feedback comprises a congratulatory message (eg, *That’s wonderful!)* whose text alternates among 5 possible phrases (Multimedia Appendix 2) and a second message that awards the user the number of points computed by our optimized gamification method (eg, *I am glad to grant you 5 points for keeping a good habit! Your total score is 49 points*). In contrast, if the user responds that they missed their chance to enact their water-drinking intention, the Good Habit Bot tells them *Okay. Keep going tomorrow!* and informs them how many points they lost and how many they have left (Multimedia Appendix 2). Afterward, the chatbot updates the user’s habit strength. Later that day, the Good Habit Bot reminds the user of their intention for the next day, and then the cycle repeats.

Critically, the chatbot computes how many points to award or take away from the user according to the optimized gamification method described previously (equation 1). One can read the number of points the chatbot we used in our experiment awarded in different situations in Figure 1 as it used the same set of parameters (ie, α=0.1; θ=0.9; *M*=13). For instance, if a user who reported having performed the desired behavior twice in the previous week enacted their intention on the first day, they earned 9 points, and their habit strength increased from




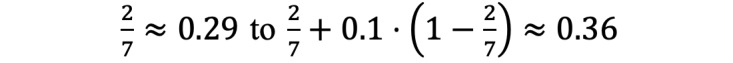




Conversely, if they failed to enact their intention, they would lose 4 points, and their habit strength would decrease to




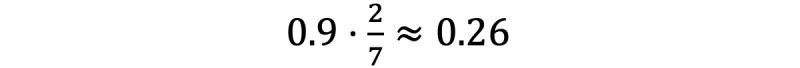




The Good Habit Bot is freely available on the Telegram messenger app, where it can be found by searching for its alias, @learn_good_habits_bot.

### Study Design

To evaluate the effectiveness of our intervention and assess the relative contributions of reminders versus optimized gamification, we ran a longitudinal between-subject experiment with 1 experimental condition with optimized gamification (*optimized gamification condition*), 1 *baseline condition*, and 1 *control condition with reminders and feedback* (Table 1). In the *optimized gamification condition*, the chatbot delivered all 4 techniques described previously, including feedback messages based on optimized gamification (Figure 1). Participants in the other 2 conditions could not gain or lose any points (no optimized gamification). They differed in whether they received feedback messages for enacting versus failing to enact the intended behavior. In the *control condition with reminders and feedback*, participants received a positive feedback message when they reported having enacted their intention (eg, *That’s wonderful!)* and a more neutral message when they reported not having done so (eg, *Okay. Keep going tomorrow!).* To create the *baseline condition*, we replaced the first control group’s positive and negative feedback message with a neutral message (*OK*) and removed the daily reminders. Participants completed the self-report measures of habit strength described in the following sections before the intervention (pretest time point), immediately after the intervention (posttest time point), and approximately 3 weeks later (follow-up). Moreover, participants reported how often they engaged in the desired behavior the week before the intervention (pretest time point) and the week after the intervention (posttest time point). Finally, participants also completed daily reports of whether they enacted their intention on each day of the intervention.

**Table 1:** Experimental conditions.

Experimental condition	Implementation intentions	Support for self-monitoring	Reminders	Feedback
Optimized gamification condition	Yes	Yes	Yes	Optimized gamification and positive vs negative text
Control condition with reminders and feedback	Yes	Yes	Yes	Positive vs negative text
Baseline condition	Yes	Yes	No	None

### Ethical Consideration

This experiment was conducted according to study protocol 401/2020BO2 approved by the Independent Ethics Commission at the Faculty of Medicine of the University of Tübingen. All data was collected and handled according to the General Data Protection Regulation of the European Union. All data has been de-identified.

### Recruitment and Reimbursement

We recruited 132 participants (n=41, 31.1% for the baseline condition; n=43, 32.6% for the optimized gamification condition; n=48, 36.4% for the control condition; n=93, 70.5% female) on the web-based research platform Prolific. Based on considerations about the cost of the study, the sample size was determined a priori so that we would retain 3x40=120 participants after an anticipated 10% of participants dropped out of the study. The requirements for participation were being a native English speaker, not having interacted with our chatbot before, and having previously completed at least 10 prolific assignments with an approval rate of at least 95%. Moreover, participants had to be aged ≥18 years. The average age of the participants was 31.5 (SD 9.9; range 19-79) years.

The study description informed participants about the study’s duration, activities, time commitment, and pay. Participants were paid £1.95 (US $2.45) for completing the onboarding survey. We informed them that the base pay for completing the remainder of the 40-day study would be £7.80 (US $9.81) and that they could earn an additional bonus of £8 (US $10.06). The description strongly recommended that only people who were already using Telegram on their smartphones should participate. Moreover, the study description informed potential participants about the potential health benefits of regular water drinking. Participants were then shown the consent form. Upon providing informed consent, participants who already had the Telegram app clicked on a link that started a conversation with the Telegram chatbot for their corresponding experimental condition. Participants who had not installed the Telegram app yet were directed to download it first.

At the end of the 40-day study, the chatbot directed participants to a second Prolific HIT where they received the announced payments contingent on their sustained active participation. All participants who completed the pretest, posttest, and follow-up measures and continued to report their daily intention enactment after the 10th day received a second payment of £15.80 (US $19.86). Participants who did not meet these criteria did not receive the second payment.

### Outcome Measures and Procedure

#### Outcome Measures

We measured the outcome variables described in the following sections.

##### Dropout

We measured whether a participant dropped out of our study using a binary variable indicating whether the participant stopped responding to all daily reports at least 3 days before the end of the study.

##### Engagement

We measured each participant’s *engagement* with our digital intervention based on the number of days on which they interacted with the Good Habit Bot.

##### Self-Report Habit Index

The Self-Report Habit Index (SRHI) [61] is a 12-item self-report measure of habit strength on a 7-point Likert scale. It comprises 3 subscales measuring the behavior’s *history of repetition*, its *automaticity*, and the extent to which it is part of the person’s *identity*. In this study, we administered the first 2 subscales. The SRHI has been found to be a 1D construct. Therefore, we averaged the scores of all items. The SRHI has been found to have high validity and very high reliability (Cronbach α of approximately 0.90; test-retest reliability: *r*=0.91).

##### Daily Intention Enactment

To measure how often each participant enacted their water-drinking intention during the intervention, we asked them the following question—*Did you accomplish your goal to drink 1 glass of water?—*on each day of the intervention. The question was asked between 30 minutes and 2.5 hours after the time at which the participant intended to drink water. Participants responded by selecting between the responses *Yes, I did!* and *No, I didn’t.* After the study, we calculated each participant’s *daily intention enactment* score by counting on how many of the 21 days of the intervention period they enacted their intention. Therefore, the daily intention enactment score could range from 0 to 21.

##### Retrospective Intention Enactment

To measure how regularly participants engaged in the intended behavior (eg, drinking a glass of water before lunch) in the weeks before and after the intervention, we asked them to answer the following question—*On how many days of the previous week did you keep the habit of drinking water?—*by selecting one of the answer choices (0, 1, 2, 3, 4, 5, or 6 days). We referred to the resulting number of days as the *retroactive intention enactment* score.

#### Procedure

We created 3 separate Prolific HITs for each of the 3 conditions of the experiment (Table 1), and each person was allowed to participate in at most one of these HITs. The experiment ran from November 11, 2021 to December 19, 2021. As illustrated in Table 2, the experiment was divided into 3 phases: the preintervention period (day 0), intervention period (days 1-21), and postintervention period (days 22-40). In the preintervention period, participants provided informed consent, completed the onboarding process, and completed the pretest. We blinded participants to the experimental manipulation by giving all participants the same information about the chatbot they were interacting with and the anticipated benefits of interacting with it. During onboarding, participants were directed to start the first conversation with the Good Habit Bot in the Telegram app on their mobile phones. In this initial conversation, the chatbot asked the participants to select a concrete daily situation in which they wanted to drink water and how much water they wanted to drink, as described previously. The pretest comprised 2 self-report measures: the *SRHI* and the *retrospective intention enactment* measure for the week before the study.

During the intervention period, each participant interacted with 1 of the 3 versions of our chatbot according to the condition they were in (Table 1). On each day of the intervention period, all 3 groups reported whether they successfully enacted their intention to drink water in the specific situation they had selected (*daily intention enactment*). At the end of the intervention period, all 3 groups completed the *SRHI* for the second time (posttest time point).

The postintervention period started with a 1-week break during which the chatbot did not communicate with the participants. Then, on day 28 (follow-up 1) and day 35 (follow-up 2), the chatbot asked participants from all 3 groups to report on how many days of the previous week (ie, the first and second week of the postintervention period, respectively) they had acted in accordance with the desired habit (*retroactive intention enactment*). Finally, on day 40, the chatbot asked all participants to complete the *SRHI* questionnaire for the third time (follow-up). Participants received 3 email reminders to resume interacting with the chatbot on the day of the first follow-up survey (day 28), the day of the second follow-up survey (day 35), and the day of the final follow-up survey (day 40).

**Table 2:** Experimental procedure.

Experimental phase and day		Optimized gamification condition	Control condition with reminders and feedback	Baseline condition
**Before the intervention**				
	Day 0	Onboarding Retrospective intention enactment SRHI^a^	Onboarding Retrospective intention enactment SRHI	Onboarding Retrospective intention enactment SRHI
**Intervention**				
	Days 1-21	Reminder, report, and feedback (optimal points+text) Daily intention enactment	Reminder, report, and feedback (text only) Daily intention enactment	Daily intention enactment
	Day 21	SRHI	SRHI	SRHI
**After the intervention**				
	Days 22-27	No reports	No reports	No reports
	Day 28	Retrospective intention enactment	Retrospective intention enactment	Retrospective intention enactment
	Days 29-34	No reports	No reports	No reports
	Day 35	Retrospective intention enactment	Retrospective intention enactment	Retrospective intention enactment
	Days 36-39	No reports	No reports	No reports
	Day 40	SRHI	SRHI	SRHI

^a^SRHI: Self-Report Habit Index.

### Data Analysis

The hypotheses and statistical analysis plan were preregistered on the internet [62]. Participants who completed 0 daily water consumption reports were excluded from all analyses apart from the dropout analysis. Other than that, all analyses were conducted on all available data from all participants who completed at least one daily water consumption report. We retained 37/41 participants from the baseline condition, 39/43 participants from the control condition with reminders and feedback, and 42/48 participants from the optimized gamification condition. All comparisons between conditions were based on the originally assigned groups. We used Poisson regression analyses for binary outcome variables. For continuous outcome variables, we used linear multilevel modeling.

## Results

There was no indication of failure of random assignment for initial habit strength, automaticity, or history of repetition (pairwise *P*>.14 in all cases).

### Dropout

As an initial step, we examined whether there was a differential dropout among the 3 conditions using a chi-square test of independence. There was no effect of condition on dropout (*χ*^2^_2_=0.7; Cramer *V*=0.07; *P*=.72).

### Engagement

The *engagement* variable was entered into a Poisson regression model with 2 dummy variables for the effects of optimized gamification and feedback and reminders. The *automaticity* and *history of repetition* scores from the SRHI before the intervention and the preintervention *retrospective intention enactment* score were entered as control variables. As shown in Table 3, optimized gamification did not increase engagement compared with the *baseline condition*. However, it appears that being in the control condition with reminders and feedback without optimal points reduced *engagement* compared with the *optimized gamification* condition (Table 3) and the *baseline* condition (incidence rate ratio=0.84, 95% CI 0.75-0.94; *P*=.002).

**Table 3:** Predicting the number of days on which participants engaged with the app (engagement) from their condition and time-1 habit-related control variables (N=126)^a^.

Predictor	Incidence rate ratio (95% CI)	*P* value
Intercept	18.88 (16.61-21.43)	<.001
Baseline vs optimized gamification	1.02 (0.91-1.14)	.72
Reminders and feedback vs optimized gamification	0.86 (0.76-0.96)	.008
Preintervention habit strength	1.03 (1.00-1.07)	.07
History of repetition	0.85 (0.79-0.92)	<.001
Automaticity	1.08 (1.01-1.16)	.03

^a^Nagelkerke *R*^2^=0.33. The treatment group with optimal points is the reference group.

### Daily Intention Enactment

The outcome variable measuring participants’ daily enactment of the desired behavior (ie, water drinking) was subjected to a Poisson regression model with the same set of predictors as for *engagement*. Critically, we found that participants in the optimized gamification condition enacted the daily intention to drink water more often (mean 14.71, SD 6.57 times) than either the participants in the baseline condition (mean 11.64, SD 5.43 times) or the participants in the control condition with reminders and feedback (mean 11.64, SD 6.38 times; Table 4). Furthermore, reminders and feedback without points did not result in more water drinking than the *baseline condition* (incidence rate ratio=1.03, 95% CI 0.90-1.17; *P*=.70).

**Table 4:** Predicting the number of days on which participants drank water (daily intention enactment) from their condition and preintervention measurements of habit-related control variables (n=118)^a^.

Predictor	Incidence rate ratio (95% CI)	*P* value
Intercept	16.02 (13.93-18.41)	<.001
Baseline vs optimized gamification	0.78 (0.69-0.89)	.001
Reminders and feedback vs optimized gamification	0.80 (0.71-0.91)	<.001
Preintervention retrospective intention enactment	1.01 (0.97-1.05)	.64
History of repetition	0.91 (0.84-0.99)	.02
Automaticity	1.07 (0.99-1.15)	.10

^a^Nagelkerke *R*^2^=0.21. The treatment group with optimal points is the reference group.

### Self-Reported Habit Strength

To test whether optimized gamification promoted habit formation, we compared the change in the SRHI self-report measures of *automaticity* and *history of repetition* from pretest to posttest to follow-up time points among the 3 experimental conditions (Figure S1 in Multimedia Appendix 3) using a multilevel model with fixed-effects predictors for time point, 2 dummy codes for the experimental condition with the *optimized gamification condition* as the reference, and all pairwise *time×condition* interactions (Tables S1 and S2 in Multimedia Appendix 3). We found that, compared with the pretest time point, both measures of habit strength were significantly higher immediately after the intervention (*P*<.001 in all cases) and at follow-up (*P*<.001 in all cases). However, these effects were no larger in the *optimized gamification condition* than in the baseline condition (*P*≥.20 in all cases) or the control condition with reminders and feedback (*P*≥.12 in all cases).

### Behavior After the Intervention

As a further test of whether the behavior change we observed during the intervention was maintained, we analyzed the number of times participants reported having enacted their intention in the week before the intervention versus the first week after the intervention and the second week after the intervention (*retrospective intention enactment*; Figure S2 in Multimedia Appendix 3) using a multilevel model with fixed-effects predictors for time point, 2 dummy codes for the experimental condition with *the optimized gamification condition* as the reference, and all pairwise *time×condition* interactions (Table S3 in Multimedia Appendix 3). We found that, compared with the week before the intervention (mean 1.8, SD 2.1 times), participants enacted the desired behavior significantly more often after the intervention (1-week follow-up: mean 5.2, SD 1.9 times, t_299_=15.21, and *P*<.001; 2-week follow-up: mean 5.1, SD 2.1 times, t_299_=14.86, and *P*<.001). However, these effects were no larger in the optimized gamification condition than they were in the baseline condition (*P*≥.18 in all cases) or the control condition with reminders and feedback (*P*≥.32 in all cases).

## Discussion

### Principal Findings

In this study, we derived a mathematical principle for designing the point systems of gamified behavior change interventions. Our proof-of-concept study suggests that this principled approach to gamifying behavior change can be beneficial. We found that our gamified behavior change chatbot fostered positive behavior change during the intervention. This is consistent with previous findings that goal setting, reinforcement, reminders, and self-monitoring are effective techniques for promoting behavior change [7,33,39,41,44].

Moreover, we found that the behavior change that occurred during the intervention was maintained in all 3 conditions. The elements that the behavior change interventions in all 3 groups shared were goal setting and self-monitoring. Therefore, goal setting and self-monitoring may be sufficient for sustained behavior change. Adding reinforcement to goal setting and self-monitoring was beneficial during the intervention, but the additional benefits of reinforcement ceased to be statistically significant (*P*≥.17 in all cases) in the week following the intervention. However, as our study had a small sample size, this apparent discrepancy could be an artifact of us having used different methods to measure behavior change during versus after the intervention. During the intervention, we measured behavior change through daily self-reports. After the intervention, we asked participants to retrospectively report on their behavior in the previous week, which is less accurate because of participants’ fallible memory, and complete self-report questionnaires about their perceived habit strength, which are less objective than reports on behavior. Consistent with the interpretation that our study had insufficient statistical power for detecting retention effects, the measures we used to assess the maintenance of behavior change consistently showed a nonsignificant trend in favor of optimized gamification (Figures S1 and S2 in Multimedia Appendix 3). Moreover, previous studies have found that gamification-induced behavior change can persist over extended periods [63].

### Limitations

From a theoretical perspective, the main limitation of our study is that it did not compare the effectiveness of the points computed by our optimized gamification method with alternative point schemes. Previous work has found optimized gamification to be more effective than several heuristic methods for designing point systems in contexts in which people choose among several activities [51]. However, the decisions that people face in behavior change applications appear to be simpler. Therefore, it remains unclear how much of the beneficial effects of optimized gamification on behavior change are specific to optimized gamification. Relatedly, it remains unclear which property of the points generated through optimized gamification is responsible for their effects on behavior change. Future work could address these questions by comparing optimized gamification of behavior change with simpler, alternative feedback mechanisms such as always awarding the same number of points or a streak-based point system.

However, we did evaluate optimized gamification against rewarding each enactment of the desired behavior using the same positive feedback message and punishing each failure to enact the desired behavior using the same negative feedback message (control condition with reminders and feedback). From a reinforcement learning perspective, this condition is equivalent to always awarding the same number of points when the behavior is enacted and always deducting the same number of points when the user fails to enact the behavior. We found that optimized gamification is more effective than this alternative feedback mechanism. This suggests that optimized gamification might be more effective than awarding the same number of points for each instance of the desired behavior. However, whether this interpretation is correct remains to be tested.

We illustrated the application of the general framework of optimized gamification to behavior change using a simple model of habit formation, which assumes that the user will indefinitely maintain the good habit once it has been established. This assumption is highly simplistic. In reality, maintenance is far from automatic. On the contrary, people may experience backsliding, and the strength of the habit may continue to wax and wane [64]. This could be captured by letting the process of deciding whether to perform the behavior continue indefinitely until the user dies. In this way, lapses could occur at any time and weaken the habit no matter how strong it is. In such a model, the health benefits of the behavior could be modeled explicitly in terms of its effects on a state variable that models the user’s health status. Refining our method’s model in this way would reduce the reinforcement for engaging in the behavior when the habit is weak and increase it when the habit is strong. This may make our method even more effective.

From an empirical perspective, the main limitation of the work presented in this paper is the relatively small sample size of our field experiment. Given that we collected <30 complete data sets per condition, the power of some of the statistical tests is relatively low. Therefore, the absence of significant differences in self-reported habit strength and retroactive intention enactment after the intervention does not provide strong evidence against the maintenance of the benefits of optimized gamification. Moreover, given that habit formation can take a very long time, our intervention may have been too short to fully capture the effects of the 3 different interventions on habit formation.

One flaw in our experiment was that some of the chatbot’s messages were not worded in perfect, idiomatic English (Multimedia Appendix 2). We think that it is unlikely that our participants misunderstood any of the messages. However, it is possible that participants would have taken the messages more seriously if all of them had been written in perfect, idiomatic English. Another minor limitation of our chatbot is that its users started with a score of 0. Thus, if they failed to enact the desired behavior, their score fell to negative values, which might be demotivating. Therefore, future versions of our chatbot will award users a number of points (eg, 20) for setting the intention to build a good habit.

Finally, another weakness of our study design is that our intervention sought to strengthen water drinking in healthy people. Therefore, we cannot draw conclusions about the potential utility of our chatbot for clinical populations for which developing a healthy water-drinking habit might be crucial [3,4]. Moreover, it remains unclear whether our findings can be generalized to other habits that are more vital to people’s health. In addition, our study was not specifically about water drinking as a weight loss strategy as only some participants tied water drinking to their meals. However, we introduced a general method that can be used to improve digital interventions for many critical behavior change applications.

### Comparison With Prior Work

This study builds on previous work on optimized gamification [51]. Optimized gamification has been previously applied to help people decide what to work on [51] and which goals to set [52,55,56,65]. Moreover, optimized gamification has also been applied to help people stay focused on their work [58]. However, the work presented in this paper is the first application of optimized gamification to support habit formation. Moreover, it is one of the first real-world applications of optimized gamification as most previous work was confined to controlled laboratory experiments.

As our chatbot combined 4 established behavior change techniques (ie, goal setting, reminders, support for self-monitoring, and reinforcement), its design and effectiveness are therefore consistent with several extant theories of behavior change [66]. In particular, our optimized gamification method is consistent with behavior change methods that acknowledge the importance of providing positive feedback on improvements in behavior [28,66-69].

The most similar gamified digital intervention for behavior change that we are aware of is the SMS text messaging–based WalkIT intervention for promoting physical exercise [7,33,70]. Participants of the WalkIT trial received SMS text messages with daily physical exercise goals. Physical exercise (walking) was measured using the accelerometers of their smartphones and reported to a server. Participants received feedback on whether they met the exercise goal via an SMS text message that included points that were converted into money. Depending on the stage of the experiment, the number of points for achieving a goal was either constant or determined at random. The number of points awarded for failing to achieve a goal was 0. In contrast, our optimized gamification method provides a principled way to choose the exact number of points that a person should be awarded for meeting their daily goal or lose for failing to meet that goal. Therefore, our method could be used to enhance the WalkIT intervention with a more principled way of choosing the number of points depending on the user’s history of successful and unsuccessful goal achievement. Conversely, WalkIT has many sophisticated features that go beyond the chatbot we introduced here. This includes an algorithm for adaptive goal setting, automatically delivered financial incentives, and an evidence-based sequence of different reward schedules that differ in the probability that goal achievement will be rewarded and whether the magnitude of the reward will be fixed or random. Consistent with our finding that the more performance-contingent feedback of the optimized gamification condition was more effective than less informative feedback or no feedback, the WalkIT studies found that immediate, behavior-contingent reinforcement was more effective in promoting behavior change than delayed, behavior-independent reinforcement.

Another gamified digital intervention for supporting behavior change is Habitica. Habitica embeds working through one’s to-do list into a role-playing game in which the user’s character can earn points by completing their daily to-dos. The points serve as an in-game currency that the player can use to buy weapons and armor for their avatar. Conversely, when the user does not complete a daily to-do, they lose points. As far as we can tell, the developers of Habitica chose the number of points the user gains for completing a to-do and the number of points they lose for failing to complete a daily to-do somewhat arbitrarily based on their intuitions. A recent study found that only 49% of Habitica’s users rate its rewards as (rather) appropriate and that most experience counterproductive effects of Habitica’s approach to gamification [17]. Given that optimized gamification was effective in our study and in previous studies, it is possible that redesigning Habitica’s point system according to optimized gamification could alleviate some of the counterproductive effects of their users’ experience.

The method introduced in this study mitigates the adverse effects of temporal discounting on people’s health behavior [26]. Its approach is to add immediate rewards that are aligned with the behavior’s long-term consequences for the user’s health; that is, optimized gamification redesigns the decision environment so that people’s shortsighted biases lead to decisions that are good for them in the long run [51]. Recent work on this topic introduced a computational model of intertemporal choice and applied it to compute personalized incentives for helping people more patiently work toward obtaining a larger reward later instead of abandoning the project in favor of a smaller immediate reward [71,72]. Similar to optimized gamification, their approach uses an MDP framework. However, their problem formulation and solution are different. The main difference lies in the application area. Sukumar et al [71] focused on canonical delay-of-gratification tasks, whereas we modeled habit formation. They tested their approach in online experiments in which participants played a *queue waiting game* and found that personalized incentives can increase people’s patience while waiting in a simulated queue. In contrast, we conducted a field experiment on behavior change in which the incentives motivated people to act more farsightedly in the real world. Despite this critical difference, investigating whether modeling and measuring individual differences can be used to make optimized gamification more effective is an exciting direction for future work. Moreover, a computational model such as the one proposed by Sukumar et al [71] could be used to simulate the effects of alternative incentive schemes.

Previous work has found that drinking water before meals is an effective weight loss strategy for adults with obesity [3,4]. In the randomized controlled trial by Parretti et al [4], participants were instructed to use the water-drinking strategy in a face-to-face weight loss consultation. They did not receive any additional support in implementing this strategy. The chatbot we developed could be used to augment those weight loss consultations with a digital tool that helps people follow through on their resolutions. Alternatively, an appropriately adapted version of our chatbot could be used as a highly scalable, low-cost alternative to face-to-face weight loss consultations.

### Conclusions

In conclusion, optimized gamification is a practically helpful theoretical principle for designing the point systems of (digital) behavior change tools and interventions. It can be implemented in just a few lines of code, and the point values can be computed instantaneously. It can be applied to improve or augment many existing (digital) behavior change interventions and can also be used to create new ones. Thus, optimized gamification can help tackle many challenging behavior change problems using scalable digital interventions. Testing whether, when, and how optimized gamification can make a positive difference in critical practical applications is an exciting direction for future research. A crucial next step will be to test our point system against simpler heuristic point systems for supporting behavior change. Moreover, our chatbot can be extended to support various health behaviors and other forms of positive behavior change.

## Appendix

Multimedia Appendix 1 An optimal feedback method for accelerating positive behavior change.

Multimedia Appendix 2 Details about the Good Habit Bot.

Multimedia Appendix 3 Details about the statistical results and supplementary analyses.

## Abbreviations

MDP: Markov decision process
SRHI: Self-Report Habit Index

## Data Availability

The data generated in this study, the R scripts used to analyze the data, and the MATLAB (MathWorks) implementation of the optimized gamification method are available on the project’s Open Science Framework repository [73].
