# Supplementary material for: Gamification of Behavior Change: Mathematical Principle and Proof-of-Concept Study
Source: JMIR Serious Games. 2024 Mar 22;12:e43078. doi: 10.2196/43078 (PMC10998180; doi:10.2196/43078)
Supplement: Multimedia Appendix 1 [file games_v12i1e43078_app1.docx]

# Multimedia Appendix 1. [An optimal feedback method for accelerating positive behavior change.](https://games.jmir.org/api/download?filename=7602e3ff08da6cd5c4dda95c6947c86c.docx&alt_name=43078-683322-3-SP.docx)

We have modelled behavior change as an MDP $M=(\mathcal{S}_{\mathrm{habit}}\mathcal{,A,}T,r,s_{0})$, where $\mathcal{S}_{\mathrm{habit}}=[0,1]$ is the set of possible habit strengths, the possible actions $\mathcal{A}=\{1,0\}$ are to either enact the desired behavior or not, the transition function $T$ is a deterministic model of habit formation, the rewards $r$ encodes the effort of enacting the behavior and the health benefits of having established it as a habit, and the initial state $s_{0}$ is the initial strength of the person’s habit. Moreover, we will refer to the value of $s_{\mathrm{habit}}$ after $t$ actions as $s_{t}$.

If the person always enacts the desired behavior, the distance of the habit strength $s_{\mathrm{habit}}$ from $1$ decays exponentially with the number of time steps. So, for $\delta_{t}=1-s_{t}$, we get $\delta_{k+t}=\delta_{k}\cdot\left( 1-\alpha\right)^{t}$. Under these conditions the sum of the rewards obtained by always enacting the desired behavior is

$$V\left( s_{k} \right)=\rho-\delta_{k}\cdot\sum_{i=1}^{n\left( s_{k};\theta\right)} \left( 1-\alpha\right)^{i-1}=\rho-\delta_{k}\cdot\frac{{1-\left( 1-\alpha\right)}^{n\left( s_{k};\theta\right)}}{\alpha},$$

where $n(s_{k};\theta)$ is the number of times that the behavior has to be performed until the terminal state is reached.

Therefore, the feedback for taking the action is $f\left( s_{k},1 \right)=V^{\star}\left( s_{k+1} \right)-V^{\star}\left( s_{k} \right)=\delta_{k}\cdot\sum_{i=1}^{n\left( s_{k};\theta\right)} \left( 1-\alpha\right)^{i-1}-\delta_{k}\cdot\left( 1-\alpha\right)\cdot\sum_{i=1}^{n\left( s_{k}+1;\theta\right)} \left( 1-\alpha\right)^{i-1}=\delta_{k}\cdot\left( \sum_{i=1}^{n\left( s_{k};\theta\right)} \left( 1-\alpha\right)^{i-1}-\sum_{i=1}^{n\left( s_{k};\theta\right)-1} \left( 1-\alpha\right)^{i} \right)=\delta_{k}\cdot\left( \sum_{i=1}^{n\left( s_{k};\theta\right)} \left( 1-\alpha\right)^{i-1}-\sum_{i=2}^{n\left( s_{k};\theta\right)} \left( 1-\alpha\right)^{i-1} \right)=\delta_{k}\cdot\left( 1-\alpha\right)^{0}=\delta_{k}=1-s_{k}$.

The feedback for not doing the action is

$$f\left( s_{k},0 \right)=\delta_{k}\cdot\frac{{1-\left( 1-\alpha\right)}^{n\left( s_{k};\theta\right)}}{\alpha}-\left( \delta_{k}+\alpha\cdot s_{k} \right)\cdot\frac{{1-\left( 1-\alpha\right)}^{n\left( s_{k}\cdot(1-\alpha);\theta\right)}}{\alpha}$$

We can compute $n(s_{k};\theta)$ by finding the smallest value of $i$ for which $1-\delta_{k}\cdot\left( 1-\alpha\right)^{i}>\theta$. This value can be computed by rounding up the number $\frac{\ln\left( 1-\theta\right)-\ln(\delta_{k})}{\ln(1-\alpha)}$.
