# Supplementary material for: Gamification of Behavior Change: Mathematical Principle and Proof-of-Concept Study
Source: JMIR Serious Games. 2024 Mar 22;12:e43078. doi: 10.2196/43078 (PMC10998180; doi:10.2196/43078)
Supplement: Multimedia Appendix 2 [file games_v12i1e43078_app2.docx]

# Multimedia Appendix 2. Details about the Good Habit Bot.

Table 3. If-Then plans supported by the Good Habit Bot.

| Options for the IF-part of the users IF-THEN plan | 1. When I leave my bed 2. When I enter the kitchen/restaurant for lunch 3. When I am going to get my lunch 4. When I have my first bite of my lunch 5. When I see my lunch is half-empty 6. When I finish my lunch When I come back from lunch |
| --- | --- |
| Options for the THEN part of the IF-THEN plan: | I will drink …   1. 0.5 glasses 2. 1 glass 3. 1.5 glasses 4. 2 glasses   of water |

Table 4. Feedback messages of the Good Habit Bot.

|  | Did the user complete their intention? | |
| --- | --- | --- |
|  | Yes. | No. |
| Possible messages | 1. That’s great! 2. That’s wonderful! 3. I can’t be more proud of you. 4. I’m so happy for you. 5. Perfect! | Okay. Keep going tomorrow. |
| Points: | I am glad to grant you 4 points for keeping a good habit! Your total scores is 53 points. | Unfortunately, I have to make -6 points from your score. Your total score is 27 points. |
