# Supplementary material for: Gamification of Behavior Change: Mathematical Principle and Proof-of-Concept Study
Source: JMIR Serious Games. 2024 Mar 22;12:e43078. doi: 10.2196/43078 (PMC10998180; doi:10.2196/43078)
Supplement: Multimedia Appendix 3 [file games_v12i1e43078_app3.docx]

Multimedia Appendix 3. Details about the Statistical Results and Supplementary Analyses.

1.
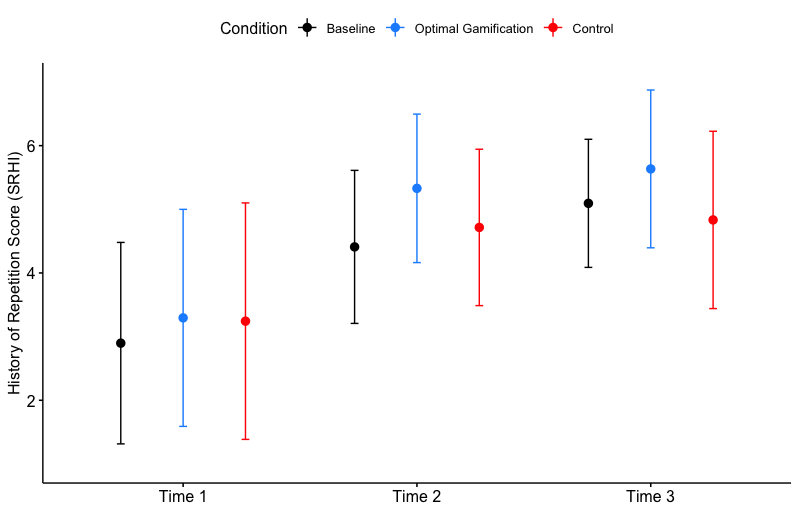


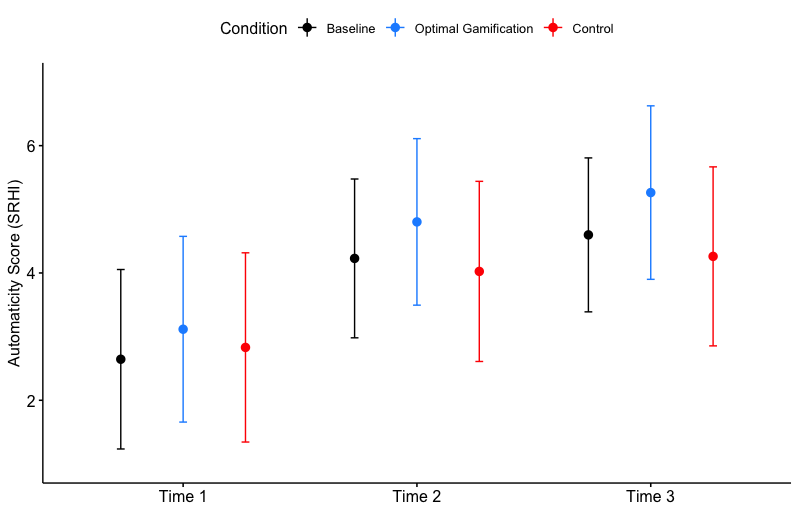


Figure S1. Self-Reported Habit Index scores for history of repetition (a) and automaticity (b) in the week before the intervention (Time 1), the first week after the intervention (Time 2), and the second week after the intervention (Time 3), respectively.


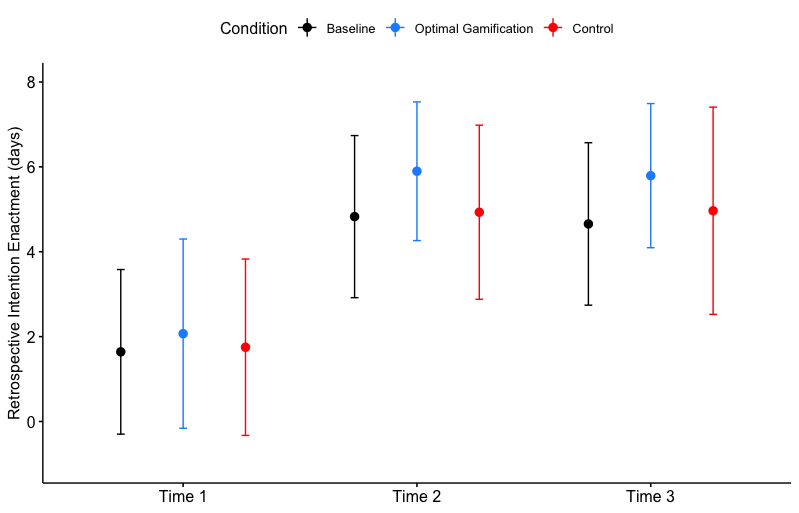


Figure S2. Retroactively reported frequency of inattention enactment in the week prior to the intervention (Time 1), the first week after the intervention (Time 2), and the second week after the intervention (Time 3).

| **Table S1. Retrospective Intention Enactment.** | | | | | | | | | | | | | |
| --- | --- | --- | --- | --- | --- | --- | --- | --- | --- | --- | --- | --- | --- |
|  | *Time Only* | | | | |  | *Time x Condition* | | | | | | |
| *Predictors* | *b* | *SE* | *CI* | *t* | *p* |  | *b* | *SE* | *CI* | | | *t* | *p* |
| (Intercept) | 1.8 | 0.18 | 1.47 – 2.18 | 10.1 | <.001 |  | 2.07 | 0.31 | 1.46 – 2.68 | | | 6.7 | <.001 |
| T2 vs. T1 | 3.5 | 0.23 | 3.02 – 3.91 | 15.2 | <.001 |  | 3.88 | 0.4 | 3.11 – 4.66 | | | 9.8 | <.001 |
| T3 vs. T1 | 3.4 | 0.23 | 2.94 – 3.83 | 14.9 | <.001 |  | 3.78 | 0.4 | 3.00 – 4.55 | | | 9.6 | <.001 |
| Baseline vs. Optimized Gamification |  |  |  |  |  |  | -0.4 | 0.45 | -1.31 – 0.45 | | | -1 | 0.34 |
| Reminders & feedback vs. Optimized Gamification |  |  |  |  |  |  | -0.3 | 0.43 | -1.17 – 0.52 | | | -0.8 | 0.46 |
| Baseline vs. Optimized Gamification x T2 vs. T1 |  |  |  |  |  |  | -0.7 | 0.56 | -1.79 – 0.42 | | | -1.2 | 0.22 |
|  |  |  |  |  |  |  |  |  |  |  |  |  |  |
| Reminders & feedback vs. Optimized Gamification x T2 vs. T1 |  |  |  |  |  |  | -0.6 | 0.56 | -1.65 – 0.54 | | | -1 | 0.32 |
|  |  |  |  |  |  |  |  |  |  |  |  |  |  |
| Baseline vs. Optimized Gamification x T3 vs. T1 |  |  |  |  |  |  | -0.8 | 0.56 | -1.86 – 0.35 | | | -1.3 | 0.18 |
|  |  |  |  |  |  |  |  |  |  |  |  |  |  |
| Reminders & feedback vs. Optimized Gamification x T3 vs. T1 |  |  |  |  |  |  | -0.4 | 0.56 | -1.51 – 0.68 | | | -0.8 | 0.45 |
|  |  |  |  |  |  |  |  |  |  |  |  |  |  |
| **Random Effects** |  |  |  |  |  |  |  |  |  | | |  |  |
| σ^2^ | 2.5 |  |  |  |  |  | 2.52 |  |  | | |  |  |
| τ_00_ | 1.68 _workerID_ | |  |  |  |  | 1.59 _workerID_ | |  | | |  |  |
| ICC | 0.4 |  |  |  |  |  | 0.39 | | |  |  |  |  |
| N | 126 _workerID_ | |  |  |  |  | 126 _workerID_ | |  | | |  |  |
| Observations | 304 |  |  |  |  |  | 304 | | |  |  |  |  |
| Marginal R^2^ / Conditional R^2^ | 0.409 / 0.647 | | |  |  |  | 0.424 / 0.647 | | | | |  |  |

| **Table S2. Automaticity** | | | | | | | | | | | |
| --- | --- | --- | --- | --- | --- | --- | --- | --- | --- | --- | --- |
|  | **Time Only** | | | | |  | **Time x Condition** | | | | |
| *Predictors* | *b* | *SE* | *CI* | *t* | *p* |  | *b* | *SE* | *CI* | *t* | *p* |
| (Intercept) | 2.9 | 0.12 | 2.64 – 3.13 | 23.2 | <0.001 |  | 3.1 | 0.21 | 2.70 – 3.53 | 14.8 | <0.001 |
| T2 vs. T1 | 1.5 | 0.13 | 1.23 – 1.74 | 11.5 | <0.001 |  | 1.7 | 0.23 | 1.22 – 2.10 | 7.38 | <0.001 |
| T3 vs. T1 | 1.8 | 0.14 | 1.57 – 2.11 | 13.4 | <0.001 |  | 2.1 | 0.24 | 1.60 – 2.55 | 8.51 | <0.001 |
| Baseline vs. Optimized Gamification |  |  |  |  |  |  | -0.5 | 0.31 | -1.07 – 0.13 | -1.5 | 0.123 |
| Reminders & feedback vs. Optimized Gamification |  |  |  |  |  |  | -0.3 | 0.29 | -0.84 – 0.31 | -0.9 | 0.371 |
| Baseline vs. Optimized Gamification x T2 vs. T1 |  |  |  |  |  |  | -0.1 | 0.33 | -0.71 – 0.58 | -0.2 | 0.845 |
|  |  |  |  |  |  |  |  |  |  |  |  |
| Reminders & feedback vs. Optimized Gamification x T2 vs. T1 |  |  |  |  |  |  | -0.4 | 0.31 | -1.04 – 0.18 | -1.4 | 0.168 |
|  |  |  |  |  |  |  |  |  |  |  |  |
| Baseline vs. Optimized Gamification x T3 vs. T1 |  |  |  |  |  |  | -0.1 | 0.35 | -0.81 – 0.54 | -0.4 | 0.698 |
|  |  |  |  |  |  |  |  |  |  |  |  |
| Reminders & feedback vs. Optimized Gamification x T3 vs. T1 |  |  |  |  |  |  | -0.5 | 0.33 | -1.18 – 0.13 | -1.6 | 0.115 |
|  |  |  |  |  |  |  |  |  |  |  |  |
| **Random Effects** |  |  |  |  |  |  |  |  |  |  |  |
| σ^2^ | 1 |  |  |  |  |  | 1.1 |  |  |  |  |
| τ_00_ | 0.97 _workerID_ | | |  |  |  | 0.90 _workerID_ | |  |  |  |
| ICC | 0.5 |  |  |  |  |  | 0.5 |  |  |  |  |
| N | 126 _workerID_ | |  |  |  |  | 126 _workerID_ | |  |  |  |
| Observations | 344 |  |  |  |  |  | 344 |  |  |  |  |
| Marginal R^2^/ Conditional R^2^ | 0.251 / 0.620 | | |  |  |  | 0.278 / 0.617 | | |  |  |

| **Table S3. History of Repetition** | | | | | | | | | | | |
| --- | --- | --- | --- | --- | --- | --- | --- | --- | --- | --- | --- |
|  | **Time Only** | | | | |  | **Time x Condition** | | | | |
| *Predictors* | *b* | *SE* | *CI* | *t* | *p* |  | *b* | *SE* | *CI* | *t* | *p* |
| (Intercept) | 3.2 | 0.13 | 2.90 – 3.41 | 24.3 | <0.001 |  | 3.3 | 0.22 | 2.86 – 3.73 | 15 | <0.001 |
| T2 vs. T1 | 1.7 | 0.14 | 1.43 – 1.96 | 12.5 | <0.001 |  | 2 | 0.23 | 1.54 – 2.46 | 8.51 | <0.001 |
| T3 vs. T1 | 2.1 | 0.14 | 1.79 – 2.35 | 14.5 | <0.001 |  | 2.3 | 0.25 | 1.80 – 2.80 | 9.05 | <0.001 |
| Baseline vs. Optimized Gamification |  |  |  |  |  |  | -0.4 | 0.32 | -1.02 – 0.23 | -1.2 | 0.214 |
| Reminders & feedback vs. Optimized Gamification |  |  |  |  |  |  | -0.1 | 0.31 | -0.67 – 0.54 | -0.2 | 0.836 |
| Baseline vs. Optimized Gamification x T2 vs. T1 |  |  |  |  |  |  | -0.4 | 0.34 | -1.11 – 0.23 | -1.3 | 0.199 |
|  |  |  |  |  |  |  |  |  |  |  |  |
| Reminders & feedback vs. Optimized Gamification x T2 vs. T1 |  |  |  |  |  |  | -0.5 | 0.33 | -1.10 – 0.18 | -1.4 | 0.156 |
|  |  |  |  |  |  |  |  |  |  |  |  |
| Baseline vs. Optimized Gamification x T3 vs. T1 |  |  |  |  |  |  | -0.1 | 0.36 | -0.77 – 0.64 | -0.2 | 0.852 |
|  |  |  |  |  |  |  |  |  |  |  |  |
| Reminders & feedback vs. Optimized Gamification x T3 vs. T1 |  |  |  |  |  |  | -0.6 | 0.35 | -1.24 – 0.12 | -1.6 | 0.105 |
|  |  |  |  |  |  |  |  |  |  |  |  |
| **Random Effects** |  |  |  |  |  |  |  |  |  |  |  |
| σ^2^ | 1.1 |  |  |  |  |  | 1.1 |  |  |  |  |
| τ_00_ | 1.05 _workerID_ | | |  |  |  | 0.99 _workerID_ | |  |  |  |
| ICC | 0.5 |  |  |  |  |  | 0.5 |  |  |  |  |
| N | 126 _workerID_ | |  |  |  |  | 126 _workerID_ | |  |  |  |
| Observations | 344 |  |  |  |  |  | 344 |  |  |  |  |
| Marginal R^2^/ Conditional R^2^ | 0.284 / 0.634 | | |  |  |  | 0.304 / 0.634 | | |  |  |
